# The Role of K_V_7.3 in Regulating Osteoblast Maturation and Mineralization

**DOI:** 10.3390/ijms17030407

**Published:** 2016-03-18

**Authors:** Ji Eun Yang, Min Seok Song, Yiming Shen, Pan Dong Ryu, So Yeong Lee

**Affiliations:** Laboratory of Veterinary Pharmacology, College of Veterinary Medicine and Research Institute for Veterinary Science, Seoul National University, 1 Gwanak-ro, Gwanak-gu, Seoul 151-742, Korea; didwldms@snu.ac.kr (J.E.Y.); gan14@snu.ac.kr (M.S.S.); sym1987@snu.ac.kr (Y.S.); pdryu@snu.ac.kr (P.D.R.)

**Keywords:** KCNQ channels, differentiation, matrix mineralization, glutamate

## Abstract

KCNQ (K_V_7) channels are voltage-gated potassium (K_V_) channels, and the function of K_V_7 channels in muscles, neurons, and sensory cells is well established. We confirmed that overall blockade of K_V_ channels with tetraethylammonium augmented the mineralization of bone-marrow-derived human mesenchymal stem cells during osteogenic differentiation, and we determined that K_V_7.3 was expressed in MG-63 and Saos-2 cells at the mRNA and protein levels. In addition, functional K_V_7 currents were detected in MG-63 cells. Inhibition of K_V_7.3 by linopirdine or XE991 increased the matrix mineralization during osteoblast differentiation. This was confirmed by alkaline phosphatase, osteocalcin, and osterix in MG-63 cells, whereas the expression of Runx2 showed no significant change. The extracellular glutamate secreted by osteoblasts was also measured to investigate its effect on MG-63 osteoblast differentiation. Blockade of K_V_7.3 promoted the release of glutamate via the phosphorylation of extracellular signal-regulated kinase 1/2-mediated upregulation of synapsin, and induced the deposition of type 1 collagen. However, activation of K_V_7.3 by flupirtine did not produce notable changes in matrix mineralization during osteoblast differentiation. These results suggest that K_V_7.3 could be a novel regulator in osteoblast differentiation.

## 1. Introduction

Voltage-gated K^+^ (K_V_) channels are one of the largest gene families among the K^+^ channel groups. K_V_ channels are known for regulating cellular electrophysiological properties in excitable cells such as neurons [1,2,3] and muscle cells [4,5,6]. In neurons and cardiac muscle cells, K_V_ channels repolarize the cell membrane after the action potential; in association with this action, K_V_ channels modulate the firing rate of the action potential, membrane stabilization, and neurotransmission [7,8]. K_V_ channels also serve as regulators in non-excitable cells. Specific K_V_ channels are expressed in most malignant cells, including those in leukemia [9], breast cancer [10,11,12,13], colon cancer [14,15,16,17], and gastric cancer [18], and they regulate cell proliferation [11,12,14,17,19,20,21,22,23], migration [16,24,25], and differentiation [22,26,27]. K_V_ channels may also affect cell volume [13,28] and cell signaling [29], leading to diverse cellular activities.

KCNQ channels are K_V_ channel members, also known as K_V_7 channels, comprising K_V_7.1 through K_V_7.5. These channels are widely distributed throughout various tissues [30]. K_V_7.1 was first identified in the heart and has been well-characterized in cardiac muscle cells [28,30]; it is also present in the inner ear epithelium [31], lung [32], and gastrointestinal tract [33]. K_V_7.2 and K_V_7.3 are mainly expressed in the central nervous system [27,34,35], usually forming a K_V_7.2/7.3 heterotetramer, which contributes M-current [36]. K_V_7.4 is present in skeletal muscle cells [22,37] and outer hair cell membrane [30], and K_V_7.5 is widely distributed in the brain [38]. Previous studies have determined the physiological role of KCNQ channels in cell proliferation, differentiation [17,22,23,37], and survival [22].

Bone is a complicated organ that continuously undergoes formative and resorptive activities, driven by osteoblasts and osteoclasts [39,40]. The development of bone depends on various extracellular signals and transcription factors to maintain its structure and homeostasis, so the microenvironment is significant for bone physiology. Sequential expressions of regulatory signals are necessary for bone cell differentiation. First, cytokines, such as bone morphogenic protein and transforming growth factor-β [41,42,43], and transcription factors, such as Runx2 [42,44,45], are required to transform a pluripotent stem cell into an osteoprogenitor cell and then into a pre-osteoblast. The pre-osteoblast then undergoes matrix mineralization, a distinctive step in osteoblast differentiation. At this stage, osteoblast-derived factors, such as osteocalcin, alkaline phosphatase, collagens, and bone sialoproteins [44,46], mediate the initiation and formation of extracellular matrix mineralization by vesicle-mediated exocytosis [47].

K_V_7 channels have been reported to regulate cell differentiation. For example, K_V_7.4 plays a role in skeletal muscle cell development [22,37], and another report indicated that K_V_7.2/7.3 was involved in neuronal differentiation through synaptic vesicle protein-mediated endo/exocytosis of neurotransmitters [27]. M-current by K_V_7 channels is controlled by multiple factors, including intracellular Ca^2+^ [30,48,49,50,51,52]. Considering the fact that Ca^2+^ is pivotal for bone homeostasis and that KCNQ channels modulate vesicular exocytosis, we explored the potential role of KCNQ channels in bone differentiation, focusing on biological mineralization of the bone matrix. First, the overall effect of K_V_ channels on human mesenchymal stem cell (hMSC) osteogenic differentiation was confirmed. We then focused on K_V_7 channel expression in osteoblast-like cells, and developed the tentative theory that K_V_7 channels, or at least K_V_7.3, may play potential roles in osteoblast differentiation through glutamatergic communication, especially for matrix maturation and mineralization.

## 2. Results

### 2.1. Effects of Tetraethylammonium, A Non-Selective Potassium Channel Blocker, on Human Mesenchymal Stem Cell Osteogenic Differentiation

**Figure 1:**
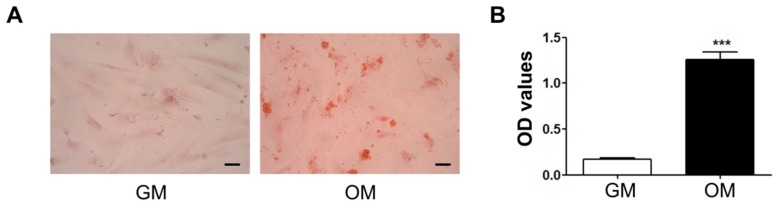

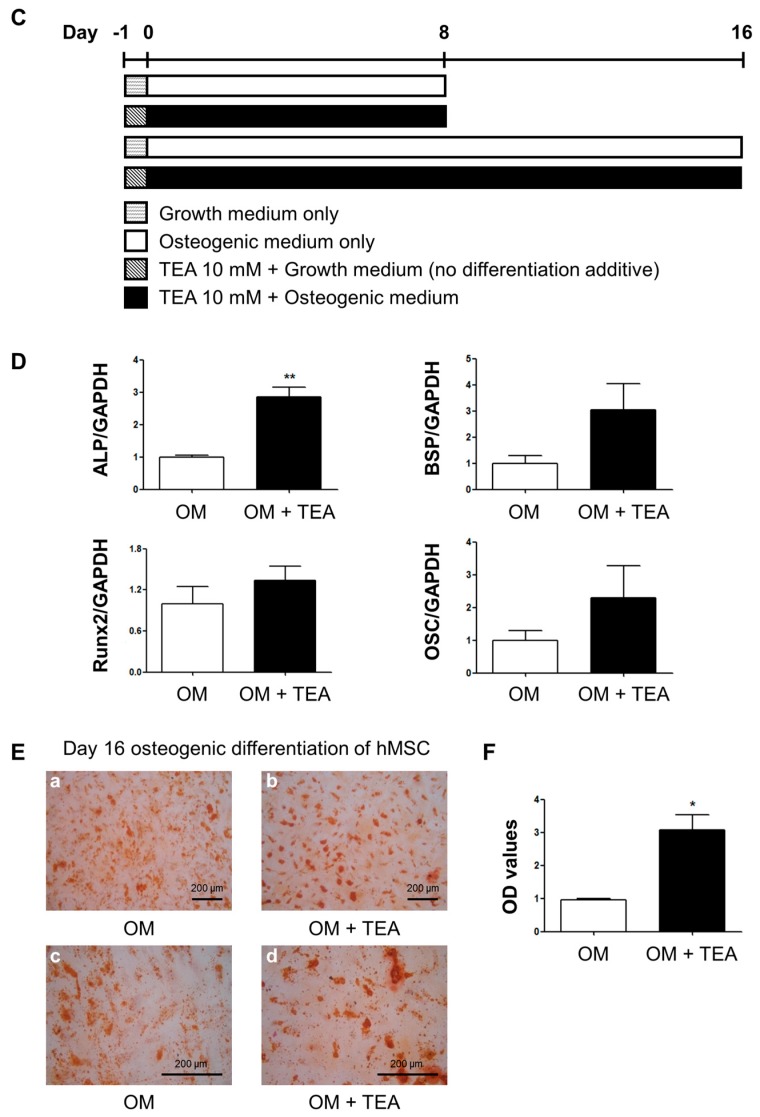
Regulation of human mesenchymal stem cell (hMSC) osteogenic differentiation by tetraethylammonium (TEA). Matrix mineralization was evaluated via Alizarin Red S staining. (**A**) Differentiated hMSCs in osteogenic-induction medium (OM) produced calcium deposits and were dyed red (right), while the cells cultured in growth medium (GM) were not stained (left). The hMSCs were differentiated for 10 days; (**B**) The OD values for Alizarin Red S staining showed that hMSCs cultured in OM had a greater calcium content than those in GM; (**C**) Scheme for the protocol of TEA treatment during hMSC osteogenic differentiation. Cells were pre-incubated with 10 mM TEA for 1 day without any differentiation additives. OM containing TEA was used to induce hMSC osteogenic differentiation; (**D**) The mRNA expressions of osteogenic differentiation markers were analyzed with qRT-PCR at day 8 of osteogenic differentiation. The mRNA expression of ALP was significantly increased. The expressions of BSP and OSC tended to increase, although not statistically significantly. There was no significant change in Runx2 expression; (**E**) Alizarin Red S staining demonstrated that hMSC osteogenic differentiation was increased by TEA. The hMSCs treated with TEA produced greater calcium contents; (**F**) The OD values demonstrated that TEA increased hMSC osteogenic differentiation. The OD values were normalized with the Alamar Blue Assay to consider remained cell numbers (*n* = 3). The data are presented as mean ± SEM. * *p* < 0.05; ** *p* < 0.01; and *** *p* < 0.005 compared to controls. Scale bar represents 200 μm. ALP: alkaline phosphatase; BSP: bone sialoprotein; OSC: osteocalcin; OD: optical density.

Bone marrow-derived human mesenchymal stem cells (hMSCs) were differentiated into the osteoblastic lineage when treated with osteogenic-induction medium (OM) for 16 days. Extracellular calcium deposits were identified via Alizarin Red S staining. The hMSCs in OM produced calcium deposits in the extracellular matrix, which represented the color red, while the cells incubated in the growth medium (GM) did not show calcium deposits (Figure 1A). The optical density (OD) values also showed that only the OM-treated hMSCs were differentiated into the osteogenic lineage (Figure 1B).

To investigate the overall effect of K_V_ channels on hMSC osteogenic differentiation, a non-selective K_V_ channel blocker, tetraethylammonium (TEA), was applied to hMSCs during differentiation. The protocol is presented in Figure 1C. Several osteoblast gene markers, such as alkaline phosphatase (ALP), bone sialoprotein (BSP), Runx2, and osteocalcin (OSC), were analyzed with quantitative RT-PCR (qRT-PCR) at day 8 of osteogenic differentiation. ALP gene expression was augmented by TEA compared to the controls (Figure 1D). TEA increased hMSC mineralization, and Alizarin Red S staining demonstrated that hMSCs treated with TEA produced more calcium deposits per unit area (Figure 1E), although fewer cells remained because of the cytotoxic effect of TEA. An Alamar Blue assay was used to determine the cytotoxicity of TEA on hMSCs (data not shown). The OD values in Figure 1F were normalized by the result of the Alamar Blue assay.

### 2.2. Expression of K_V_7 Channels in Osteoblast-Like Cell Lines, MG-63 and Saos-2

**Figure 2:**
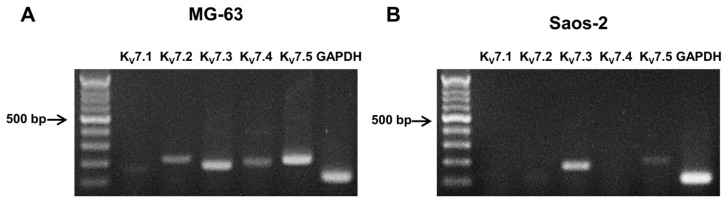
RT-PCR analysis of the K_V_7 channels in osteoblast-like cells. The PCR products using cDNA from the MG-63 (**A**) and Saos-2 cells (**B**) were electrophoresed in a 1.6% agarose gel (*n* = 5).

To identify the effect of K_V_ channels on osteogenic properties, the mRNA expression of KCNQ gene subfamilies, including K_V_7.1, K_V_7.2, K_V_7.3, K_V_7.4, and K_V_7.5 in MG-63 and Saos-2 cells, was analyzed using RT-PCR. While all K_V_7 channel subtypes were present in the MG-63 cells (Figure 2A), K_V_7.3 and K_V_7.5 were expressed in the Saos-2 cells (Figure 2B).

### 2.3. mRNA and Protein Expression and Functional Activities of Kv7 Channels during Osteoblastic Differentiation

**Figure 3:**
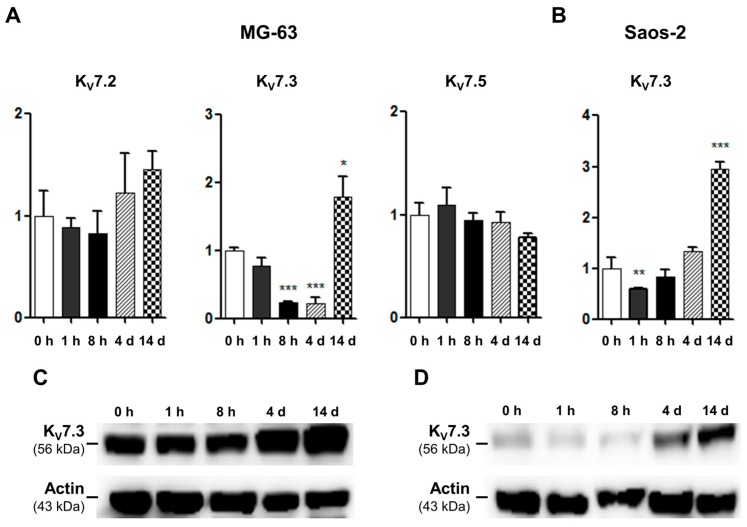
Changes in K_V_7 channel expression during osteoblastic differentiation. The relative expression levels of K_V_7.2, K_V_7.3, and K_V_7.5 during osteoblast differentiation were analyzed by qRT-PCR. (**A**) In MG-63 cells, mRNA expressions of K_V_7.2 and K_V_7.5 were not significantly changed (*n* = 3). However, K_V_7.3 transcripts decreased for 4 days after osteoblast induction, whereas, at day 14, the K_V_7.3 mRNA level was considerably increased (*n* = 4); (**B**) In Saos-2 cells, mRNA expression of K_V_7.3 was reduced 1 h after osteoblast induction, but was significantly augmented at day 14 (*n* = 4); (**C**,**D**) The expression of K_V_7.3 proteins was increased at day 4 of osteoblast induction (*n* = 3). Data are presented as mean ± SEM. * *p* < 0.05; ** *p* < 0.01; and *** *p* < 0.005. d: day.

In RT-PCR analysis, we confirmed that MG-63 cells strongly express K_V_7.2, K_V_7.3, and K_V_7.5 in MG-63 and K_V_7.3 in Saos-2 cells. We therefore examined the expression levels of these channels during osteoblast differentiation using qRT-PCR. These results demonstrated that the mRNA levels of K_V_7.2 and K_V_7.5 were not significantly changed during osteoblast differentiation in MG-63 cells (Figure 3A). In contrast, the mRNA expression of K_V_7.3 significantly decreased 8 h after osteoblast induction; however, at day 14, the mRNA level of K_V_7.3 showed a substantial increase (Figure 3A). Similarly, in Saos-2 cells, while the K_V_7.3 transcripts significantly decreased 1 h after osteoblast induction, the K_V_7.3 transcripts level increased considerably at day 14 of osteoblast differentiation (Figure 3B). We also investigated the changes in K_V_7.3 protein expression, and the western blot analysis illustrated that K_V_7.3 proteins increased at days 4 and 14 after osteoblast induction in the MG-63 and Saos-2 cells (Figure 3C,D).

**Figure 4:**
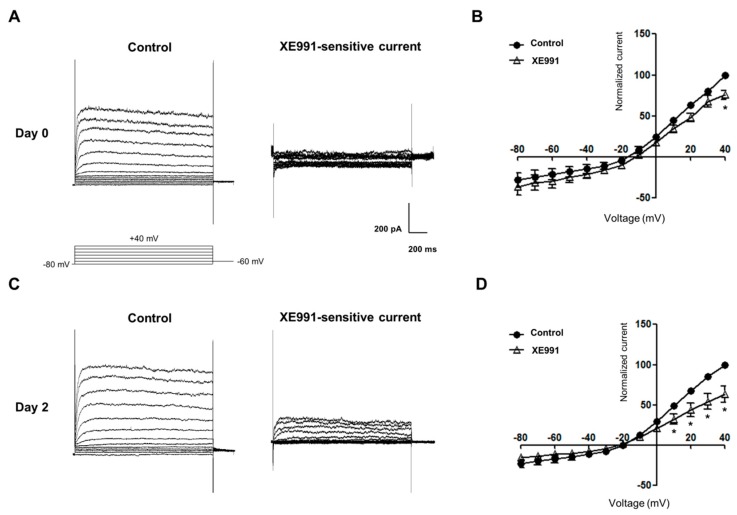

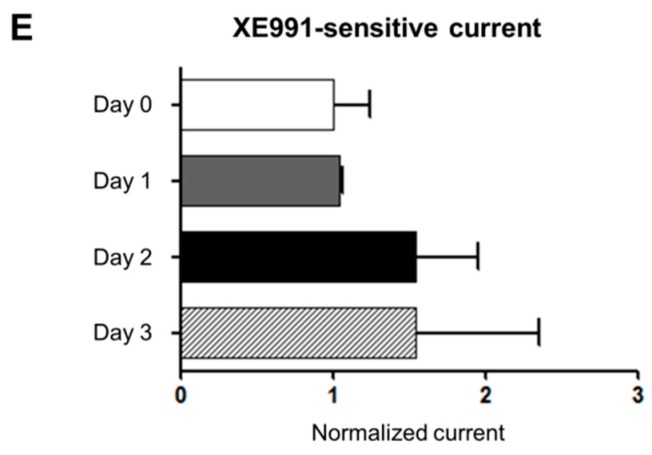
Functional characteristics of K_V_7.3 channel in MG-63 cells during osteoblast differentiation. (**A**) Representative current responses to depolarizing steps observed at day 0 of undifferentiated MG-63 cells. XE991-sensitive currents were obtained by the subtraction of the currents in controls from in the presence of XE991 (50 μM); (**B**) Current-voltage relationships in controls and in the presence of XE991 at day 0 (*n* = 3); (**C**) Representative current responses to depolarizing steps observed at day 2 of osteoblastic differentiation; (**D**) Current-voltage relationships in controls and in the presence of XE991 at day 2 (*n* = 4); (**E**) XE991-sensitive currents at days 0–4 were normalized by mean current value at day 0. At day 1 of osteoblast differentiation, normalized XE991-sensitive currents had no significant change. At days 2 and 3, the K_V_7.3 currents were increased, but not statistically significant. Data are presented as mean ± SEM. * *p* < 0.05.

Whole-cell patch clamp recordings were performed on early osteoblastic differentiation before extracellular matrix production began. These results showed that functional K_V_7.3 currents were present in MG-63 cells at days 0 and 2 of osteoblast differentiation (Figure 4A,C). Voltage-current relationships illustrated that XE991 significantly inhibited K_V_7.3 currents (Figure 4B,D). Normalized XE991-sensitive K_V_7.3 currents at days 2 and 3 were increased by 54.03% and 54.52%, respectively, but not statistically significant (Figure 4E).

**Figure 5:**
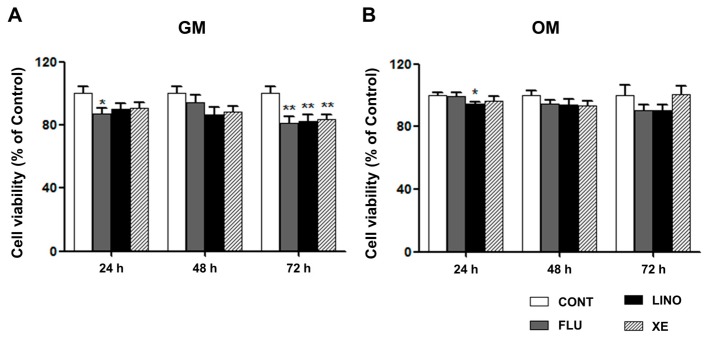
Effect of flupirtine, linopirdine, and XE991 on MG-63 cell viability. The MTT assay was performed on MG-63 cells. (**A**) MG-63 cells were incubated in growth medium (GM) with 30 μM of flupirtine, 30 μM of linopirdine, and 10 μM of XE991. At 72 h, linopirdine, XE991, and flupirtine caused notable decreases in cell viability (*n* = 16); (**B**) MG-63 cells were cultured in osteoblast-induction medium (OM) with 30 μM of flupirtine, 30 μM of linopirdine, and 10 μM of XE991. The cell viability was not significantly influenced by OM containing linopirdine, XE991, or flupirtine for 72 h of treatment (*n* = 16). The values are presented as mean ± SEM. * *p* < 0.05 and ** *p* < 0.01. CONT: non-treated controls; FLU: flupirtine; LINO: linopirdine; XE: XE991.

To confirm the effect of K_V_7.3 modulators on cell viability, the MTT assay was performed on MG-63 cells. A K_V_7 opener, flupirtine (30 μM), and K_V_7.3 blockers, linopirdine (30 μM) and XE991 (10 μM) were used. Although treatment with flupirtine attenuated cell viability at 24 h, overall cell viability was not significantly affected by treatment with flupirtine, linopirdine, and XE991 at 24 and 48 h. However, at 72 h, the K_V_7 opener and K_V_7.3 blocker-treated groups showed significant decreases in cell viability in GM (Figure 5A). To further understand the effect on cell viability during osteoblastic differentiation, we cultured MG-63 cells in OM under the same conditions, and then performed MTT assays. The results indicated that although linopirdine reduced cell proliferation at 24 h, there was no significant change in overall cell viability, when treated with OM containing the K_V_7.3 blockers or the K_V_7 opener (Figure 5B).

### 2.4. Regulation of Mineralization by K_V_7.3 Blockers or the K_V_7 Opener in MG-63 and Saos-2 Cells

**Figure 6:**
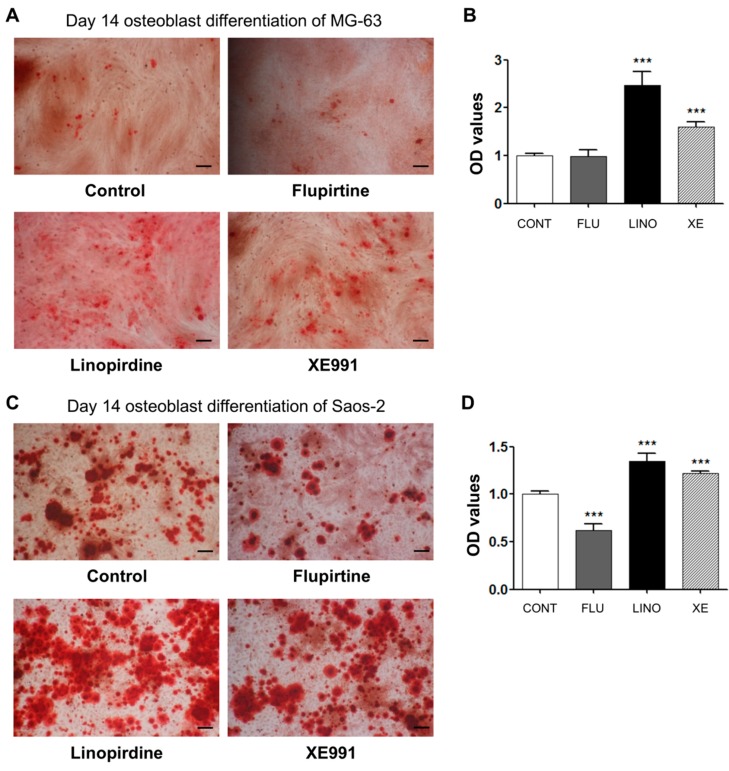
Regulation of osteoblastic differentiation by K_V_7 channel in MG-63 and Saos-2 cells. (**A**) Alizarin Red S staining data showed that 30 μM of linopirdine and 10 μM of XE991 augmented mineralization in the extracellular matrix in MG-63 cells (*n* = 10). There was no significant change in the mineralized matrix with 30 μM of flupirtine (*n* = 3); (**B**) The OD values demonstrated that linopirdine and XE991 increased the amount of calcium deposits; (**C**) Alizarin Red S staining illustrated that mineralization of Saos-2 cells was increased with 30 μM of linopirdine and 10 μM of XE991 (*n* = 8), while 30 μM of flupirtine reduced the amount of calcium deposits (*n* = 7); (**D**) The OD values are shown parallel to Alizarin Red S staining results. Data are presented as mean ± SEM. *** *p* < 0.005. Scale bar represents 100 μm. CONT: non-treated controls; FLU: flupirtine; LINO: linopirdine; XE: XE991; OD: optical density.

We confirmed the effect of the K_V_7.3 channel on calcium deposition in the extracellular matrix using Alizarin Red S staining. The K_V_7.3 blockade by linopirdine (30 μM) or XE991 (10 μM) noticeably increased mineralization in MG-63 cells. On the other hand, K_V_7 activation by flupirtine (30 μM) did not show an effect on mineralization (Figure 6A,B). These experiments were conducted with additional osteoblast-like Saos-2 cells to examine the effect of blocking K_V_7 channels on mineralization. The results showed that linopirdine (30 μM) and XE991 (10 μM) significantly promoted mineralization, whereas flupirtine (30 μM) attenuated the extent of calcium deposits of Saos-2 cells (Figure 6C,D). Therefore, the inhibition of K_V_7.3 channels augmented mineralization, facilitating osteoblast differentiation.

### 2.5. Regulation of Osteoblast Differentiation Markers by K_V_7 Channels in MG-63 Cells

**Figure 7:**
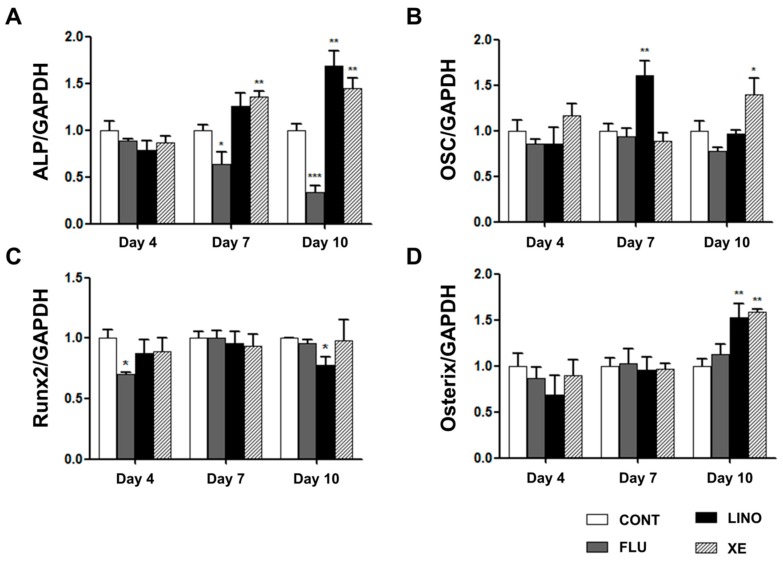
mRNA expression of osteoblastic differentiation markers in MG-63 cells. The relative mRNA expression levels of osteoblastic differentiation markers, ALP, OSC, Runx2, and osterix, were measured with qRT-PCR and normalized against glyceraldehyde 3-phosphate dehydrogenase (GAPDH) expression. (**A**) While linopirdine (30 μM) and XE991 (10 μM) increased ALP mRNA expression at days 7 and 10 of osteoblastic induction, flupirtine (30 μM) decreased ALP mRNA expression at days 7 and 10 (*n* = 3–7); (**B**) The mRNA expression level of OSC was increased by linopirdine or XE991 at days 7 and 10, respectively (*n* = 3–7); (**C**) mRNA expression of Runx2 was decreased by flupirtine at day 4 and by linopirdine at day 10 (*n* = 3); (**D**) Osterix gene expression was increased by linopirdine and by XE991 at day 10 of osteoblastic induction (*n* = 3–7). The values are presented as mean ± SEM. * *p* < 0.05; ** *p* < 0.01; and *** *p* < 0.005. ALP: alkaline phosphatase; OSC: osteocalcin; CONT: non-treated controls; FLU: flupirtine; LINO: linopirdine; XE: XE991.

We examined the mRNA expression of ALP, OSC, Runx2, and osterix using qRT-PCR. Linopirdine (30 μM) and XE991 (10 μM) significantly increased ALP gene expression at days 7 and 10 of osteoblast induction, whereas K_V_7 activation by flupirtine (30 μM) attenuated the expression of ALP (Figure 7A). Additionally, the mRNA expression of OSC was increased by linopirdine and XE991 at days 7 and 10, respectively, while flupirtine showed no significant effect on OSC expression (Figure 7B). We also identified the expression of Runx2 and osterix, which are known as essential transcription factors that promote hMSC differentiation into osteogenic lineages. Although the level of Runx2 was decreased by linopirdine at day 10, linopirdine and XE991 did not produce changes in Runx2 levels through overall osteoblast differentiation (Figure 7C). However, at day 10, blockade of K_V_7.3 by linopirdine and XE991 augmented the mRNA expression of osterix; flupirtine did not result in significant changes (Figure 7D).

### 2.6. Effect of K_V_7 Channel Modulations on Synaptic Vesicle-Related Synapsin and the Mitogen-Activated Protein Kinase (MAPK) Signaling Pathway

**Figure 8:**
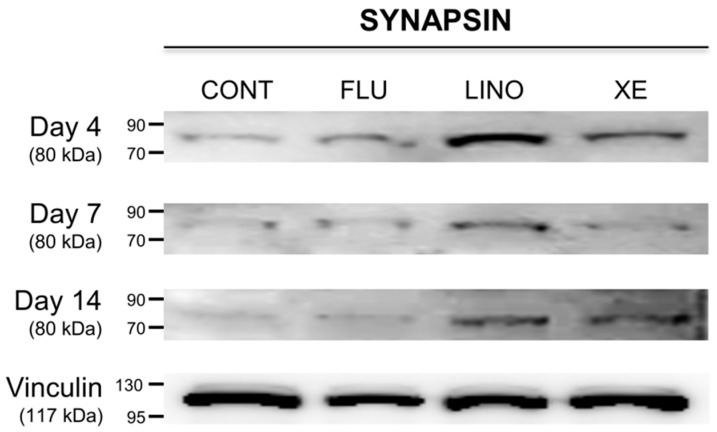
Regulation of synaptic vesicle-related protein, synapsin, by K_V_7.3 channel in MG-63 cells. Western blot analysis showed that K_V_7.3 blockade by linopirdine (30 μM) or XE991 (10 μM) increased synapsin expression during osteoblast differentiation. However, K_V_7 activation by flupirtine (30 μM) had no significant effect on the protein expression of synapsin (*n* = 3). Vinculin is used as a loading control for Western blot analysis. CONT: non-treated controls; FLU: flupirtine; LINO: linopirdine; XE: XE991.

**Figure 9:**
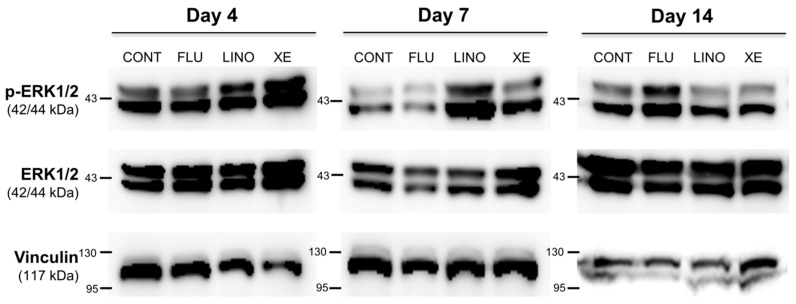
Alterations of ERK1/2 phosphorylation by the K_V_7 opener or K_V_7.3 blockers in MG-63 cells. Western blot analysis showed that, while linopirdine (30 μM) and XE991 (10 μM) increased the expression level of ERK1/2 phosphorylation at days 4 and 7 of osteoblast induction, flupirtine (30 μM) had no significant effect on the level of ERK1/2 phosphorylation. Treatment with linopirdine or XE991 showed no increase of ERK1/2 phosphorylation at day 14 of osteoblast differentiation (*n* = 3). Flupirtine augmented the expression of ERK1/2 phosphorylation at day 14 (*n* = 3). Vinculin is used as a loading control for Western blot analysis. CONT: non-treated controls; FLU: flupirtine; LINO: linopirdine; XE: XE991; ERK1/2: extracellular-signal-regulated kinase 1/2; p-ERK: ERK1/2 phosphorylation.

In previous experiments, we identified that K_V_7.3 inhibition using linopirdine or XE991 increased mineralization during osteoblast differentiation. Focusing on these results, synapsin and extracellular signal-regulated kinase 1/2 (ERK1/2) expressions were examined to verify whether K_V_7 channels were involved in vesicular exocytosis during matrix mineralization. First, the expression of the synaptic vesicle-related protein synapsin was increased by the K_V_7.3 blockers, linopirdine (30 μM) and XE991 (10 μM), whereas K_V_7 activation by flupirtine (30 μM) had no effect (Figure 8). In the same manner, while inhibition of K_V_7.3 by linopirdine or XE991 increased the ERK1/2 phosphorylation (p-ERK1/2), K_V_7 activation by flupirtine resulted in no significant changes to p-ERK1/2 levels. On the other hand, at day 14, blockade by linopirdine or XE991 produced no increase in p-ERK1/2, but flupirtine increased its expression (Figure 9).

### 2.7. Induction of Glutamate Release and Type 1 Collagen by K_V_7.3 Channels during MG-63 Osteoblast Differentiation

**Figure 10:**
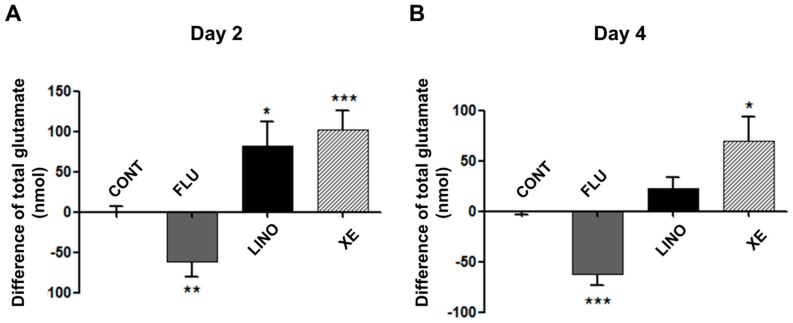
Effect of K_V_7 channel on glutamate release during osteoblastic differentiation in MG-63 cells. (**A**) On day 2, flupirtine (30 μM) significantly decreased glutamate release after inducing osteoblastic differentiation (*n* = 6). However, linopirdine (30 μM) or XE991 (10 μM) caused significantly increased glutamate release (*n* = 5); (**B**) On day 4, flupirtine also caused decreased glutamate release (*n* = 5), whereas XE991 notably increased the extracellular glutamate (*n* = 7). Linopirdine augmented the amount of glutamate, but not statistically significantly (*n* = 8). The values are presented as mean ± SEM. * *p* < 0.05; ** *p* < 0.01; and *** *p* < 0.005. CONT: non-treated controls; FLU: flupirtine; LINO: linopirdine; XE: XE991.

To investigate the effect of K_V_7 channels on glutamate release during osteoblastic differentiation, we measured the amount of extracellular glutamate secreted by osteoblasts. Figure 9 demonstrates the difference of total glutamate amounts between the control groups and the groups treated with flupirtine (30 μM), linopirdine (30 μM), and XE991 (10 μM). The K_V_7 activation by flupirtine significantly reduced the amount of glutamate release on days 2 and 4 after inducing osteoblast differentiation (Figure 10A,B). In contrast, the K_V_7.3 blockade by linopirdine or XE991 increased the amount of extracellular glutamate (Figure 10A,B).

**Figure 11:**
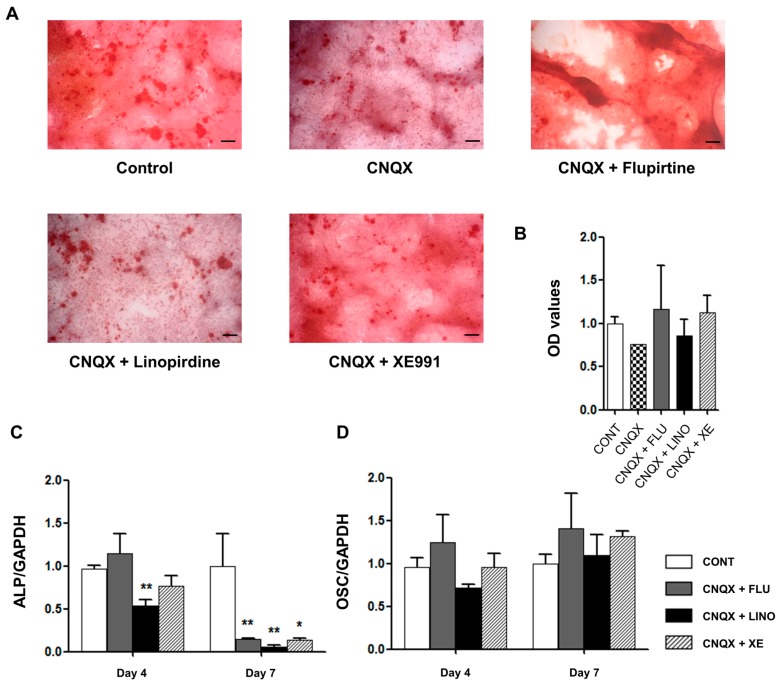
Suppressive effect of CNQX (6-cyano-7-nitroquinoxaline-2,3-dione), an AMPA (α-amino-3-hydroxy-5-methyl-4-isoxazolepropionic acid)/kainite receptor antagonist, on osteoblastic differentiation promoted by K_V_7.3 blockers. (**A**) Alizarin Red S staining showed that CNQX (50 μM) co-applied with flupirtine (30 μM), linopirdine (30 μM), or XE991 (10 μM) produced amounts ofcalcium deposits similar to those of the controls (*n* = 3). (**B**) The OD values are shown parallel to Alizarin Red S staining results. The relative mRNA expression levels of osteoblastic differentiation markers, including ALP and OSC, were measured with qRT-PCR and normalized against GAPDH (glyceraldehyde 3-phosphate dehydrogenase) expression (*n* = 3). (**C**) At day 4, CNQX treatment with XE991 significantly reduced the ALP mRNA expression. At day 7, CNQX with flupirtine, linopirdine, or XE991 attenuated the ALP level. (**D**) CNQX treatment with flupirtine, linopirdine, or XE991 showed no significant changes in the OSC levels. Data are presented as mean ± SEM. * *p* < 0.05 and ** *p* < 0.01. Scale bar represents 100 μm. CONT: non-treated controls; FLU: flupirtine; LINO: linopirdine; XE: XE991; ALP: alkaline phosphatase; OSC: osteocalcin; OD: optical density.

**Figure 12:**
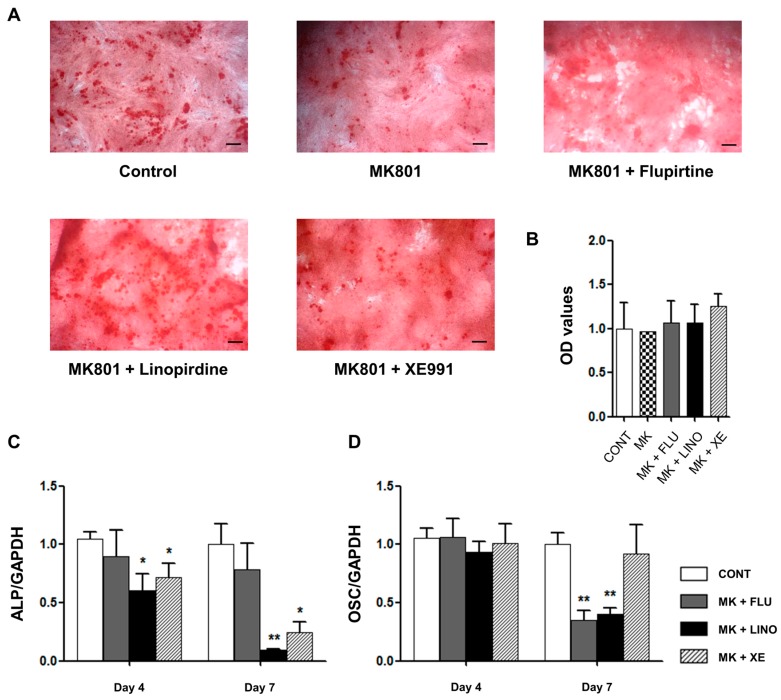
Suppressive effect of MK801, an NMDA (N-methyl-D-aspartate) receptor antagonist, on osteoblastic differentiation promoted by K_V_7.3 blockers. (**A**) Alizarin Red S staining showed that MK801 (50 μM) treatment co-applied with flupirtine (30 μM), linopirdine (30 μM), or XE991 (10 μM) produced amounts of calcium deposits similar to those of the controls (*n* = 3); (**B**) The OD values are shown parallel to Alizarin Red S staining results. The relative mRNA expression levels of osteoblastic differentiation markers, including ALP and OSC, were measured with qRT-PCR and normalized against GAPDH (glyceraldehyde 3-phosphate dehydrogenase) expression (*n* = 3); (**C**) At days 4 and 7, MK801 treatment with linopirdine or XE991 attenuated ALP mRNA expression; (**D**) At day 7, MK801 treatment with flupirtine or linopirdine significantly reduced OSC mRNA expression. Data are presented as mean ± SEM. * *p* < 0.05 and ** *p* < 0.01. Scale bar represents 100 μm. CONT: non-treated controls; MK: MK801; FLU: flupirtine; LINO: linopirdine; XE: XE991; ALP: alkaline phosphatase; OSC: osteocalcin; OD: optical density.

**Figure 13:**
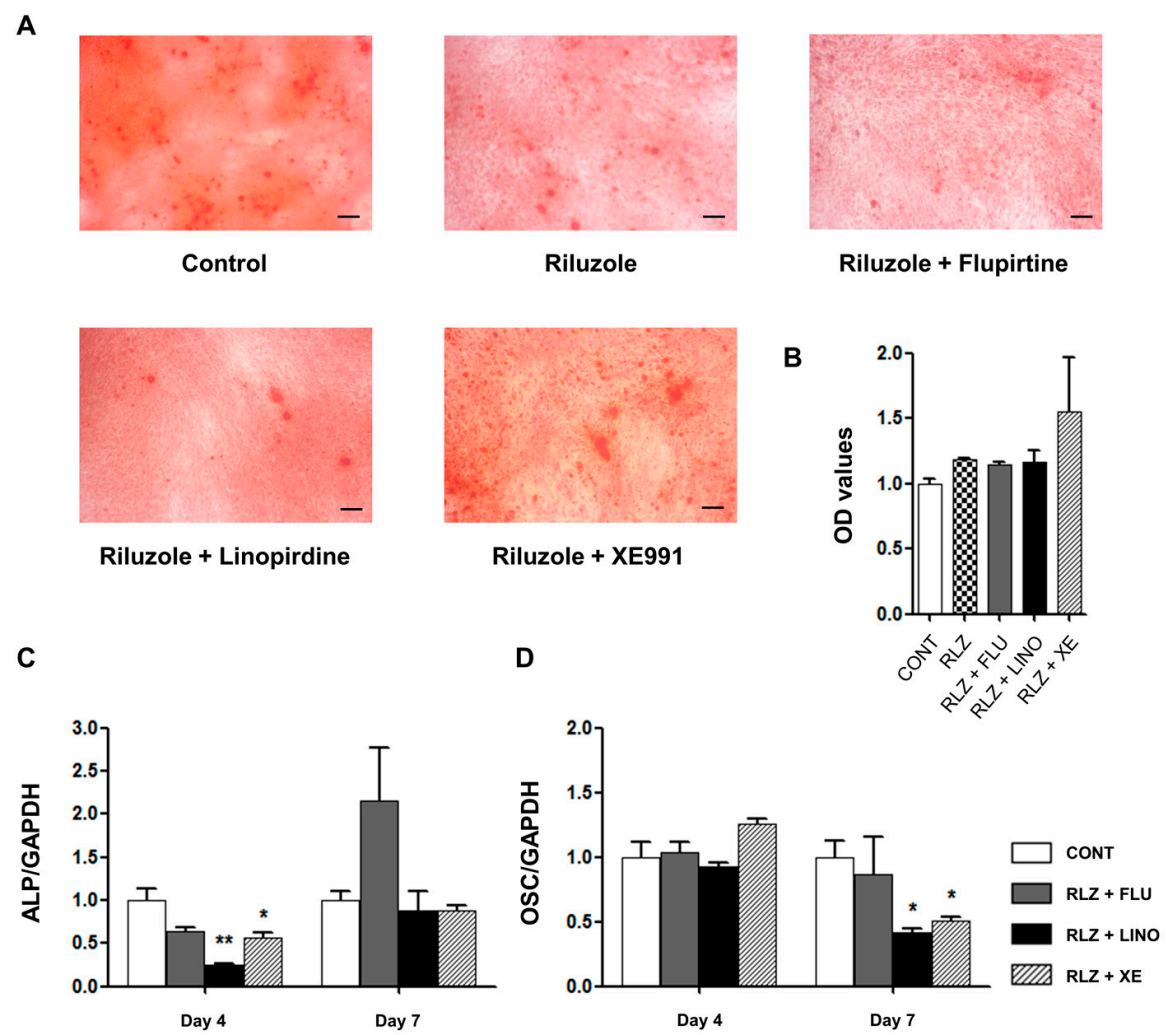
Counter-effect of riluzole, a glutamate release inhibitor, on osteoblastic differentiation promoted by K_V_7.3 blockers. (**A**) Alizarin Red S staining showed that riluzole (30 μM) co-applied with flupirtine (30 μM), linopirdine (30 μM), or XE991 (10 μM) produced mineralization levels similar to those of the controls (*n* = 3); (**B**) The OD values are shown parallel to Alizarin Red S staining results. The relative mRNA expression levels of osteoblastic differentiation markers, including ALP and OSC, were measured with qRT-PCR and normalized against GAPDH (glyceraldehyde 3-phosphate dehydrogenase) expression (*n* = 3); (**C**) At day 4, riluzole treatment with linopirdine or XE991 reduced the mRNA expression of ALP. At day 7, there was no significant change in ALP levels, although riluzole treatment with flupirtine had a tendency to increase these levels; (**D**) At day 7, riluzole treatment with linopirdine or XE991 reduced the OSC mRNA expression. Data are presented as mean ± SEM. * *p* < 0.05 and ** *p* < 0.01. Scale bar represents 100 μm. CONT: non-treated controls; RLZ: riluzole; FLU: flupirtine; LINO: linopirdine; XE: XE991; ALP: alkaline phosphatase; OSC: osteocalcin; OD: optical density.

To examine whether the augmentation of glutamate release by K_V_7.3 blockers can directly promote osteoblastic differentiation, CNQX (6-cyano-7-nitroquinoxaline-2,3-dione), an AMPA α-amino-3-hydroxy-5-methyl-4-isoxazolepropionic acid)/kainite receptor antagonist, and MK801, an NMDA (*N*-methyl-d-aspartate) receptor antagonist, were used. Mineralized deposits augmented by K_V_7.3 blockers were reduced by co-application of CNQX (50 μM) (Figure 11A,B) and MK801 (50 μM) (Figure 12A,B). Additionally, CNQX treatment with linopirdine or XE991 attenuated the expression of ALP at days 4 and 7 (Figure 11C), whereas the OSC level was not affected (Figure 11D). Treatment with MK801 and K_V_7.3 blockers reduced the ALP levels at days 4 and 7 (Figure 12C), and linopirdine and flupirtine with MK801 attenuated the OSC levels at day 7 (Figure 12D). Riluzole, a glutamate release inhibitor, was also applied. Riluzole (30 μM) co-applied with linopirdine (30 μM) or XE991 (10 μM) counteracted the promotive effects of linopirdine and XE991 alone on extracellular mineralization (Figure 13A,B). The mRNA expression of ALP was decreased by riluzole treatment with K_V_7.3 blockers, including linopirdine or XE991, at day 4 (Figure 13C), while riluzole with linopirdine or XE991 attenuated the OSC level at day 7 (Figure 13D). However, co-treatment with riluzole and the K_V_7 opener—flupirtine (30 μM)—caused no significant changes in the matrix mineralization (Figure 13A,B) or in the expression of ALP and OSC (Figure 13C,D). These results suggested that glutamate receptor antagonism or glutamate release inhibition counteracted the promotive effects of linopirdine and XE991 on matrix mineralization during osteoblastic differentiation.

**Figure 14:**
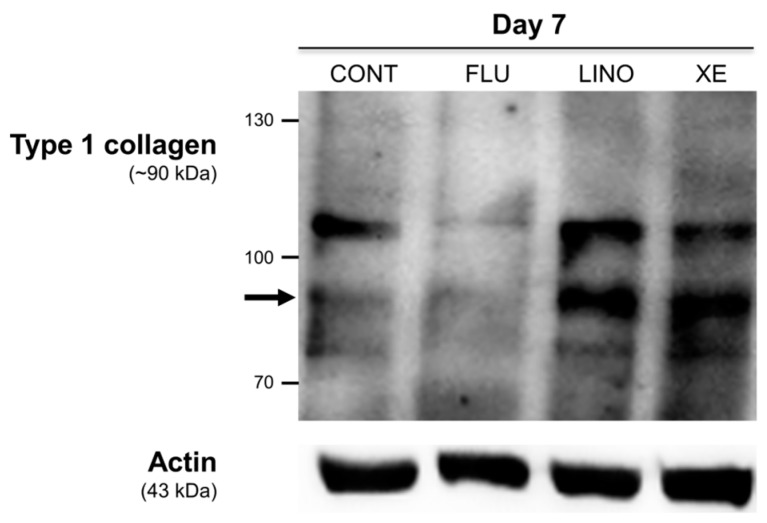
Induction of intracellular type 1 collagen by K_V_7 channel during osteoblast differentiation in MG-63 cells. Western blot analysis demonstrated that 30 μM of linopirdine or 10 μM of XE991 considerably increased the expression of type 1 collagen on day 7 of osteoblastic induction, while 30 μM of flupirtine attenuated type 1 collagens. CONT: non-treated controls; FLU: flupirtine; LINO: linopirdine; XE: XE991.

To further understand the role of K_V_7.3 channels in extracellular matrix mineralization, the expression of intracellular type 1 collagens, which are involved in matrix maturation during osteoblast differentiation, was investigated. The blockade of K_V_7.3 by linopirdine (30 μM) or XE991 (10 μM) augmented the level of type 1 collagens on day 7 of osteoblastic differentiation. The K_V_7 activation by flupirtine (30 μM) decreased the expression of type 1 collagens (Figure 14).

## 3. Discussion

The present study demonstrates that K_V_7.3 channels have potential effects on osteoblast differentiation. Our results showed that blockade of K_V_7.3 channels by linopirdine or XE991 remarkably increased extracellular mineralization during osteoblast differentiation. In contrast, activation of K_V_7.3 channels by flupirtine did not produce significant changes. The osteoblast marker genes, ERK1/2 phosphorylation, synaptic-vesicle protein, synapsin, glutamate signals, and type 1 collagens were also involved in the process of osteoblast differentiation.

Several studies have reported that ion channels, including voltage-gated calcium channels [53] and ether-à-go-go 1 channels [54,55,56], are involved in cell proliferation in MG-63 or Saos-2 cells. However, ion channels are not widely known for regulating osteoblast differentiation. The overexpression of chloride channel-3 enhances osteogenic differentiation through Runx2-mediated ALP, BSP, and OSC genes by regulating intracellular pH in MC3T3-E1 primary mouse osteoblasts [57]. Chloride intracellular channel 1 also induces osteoblast marker genes by hyperpolarization of the mitochondrial membrane in C3H10T1/2 mouse embryonic mesenchymal cells [58]. Among the K^+^ channels, the inwardly rectifying potassium channel Kir2.1 was recently reported to regulate osteoblastic differentiation, and blockade of Kir2.1 attenuated matrix mineralization, suggesting that Kir2.1 channel is critical during osteoblastogenesis [59]. In contrast, blockade of large-conductance potassium channels with TEA increased mineralization in human primary osteoblasts [60], and hSlo potassium channels are reported to regulate bone remodeling by responding the mechanical loads in MG-63 and CAL72 osteosarcoma cells [61].

Most studies concerning osteoblast differentiation have focused on mesenchymal stem cells (MSCs) [58] or primary osteoblasts [57,60]. Our study, however, used MG-63 and Saos-2 osteoblast-like cells, which have distinct characteristics compared to MSCs or primary osteoblasts [62,63]. MG-63 is the immature state of the osteoblast, and the expressions of OSC, BSP, and Runx2 osteoblast markers are relatively lower than those of primary osteoblasts, with inconsistencies in mineralization [62,63]. Unlike MG-63, Saos-2 mature osteoblast cells have high levels of ALP enzymatic activity and a strong capability to create a calcified matrix [62], which was shown in our experiments. Another report showed dissimilar patterns of osteoblast genes, such as Runx2, in the osteoblast differentiation of Saos-2 cells in comparison to primary osteoblasts [64]. This is because MSCs can differentiate into pre-osteoblasts, immature osteoblasts, and mature osteoblasts sequentially [44], but the Saos-2 cells are the mature state of the osteoblast. Hence, Saos-2 and MG-63 cells undergo osteoblastic differentiation from the immature or mature state of osteoblasts [62], making it possible to concentrate on osteoblast maturation and matrix mineralization [65].

To date, little is known about the role of K_V_ channels in MG-63 and Saos-2 cells. Although several studies confirmed the existence of TEA-sensitive [66] or K_V_2.1-related outward K_V_ currents [67], and the existence of K_V_7 channels has been demonstrated in MG-63 cells [23], the physiologic functions of K_V_7 channels in MG-63 and Saos-2 cells are not widely known. In the present study, we identified the mRNA and protein expression of K_V_7.3 channels in MG-63 and Saos-2 cells. Although we also demonstrated that functional K_V_7.3 currents were present in MG-63 cells, the K_V_7.3 currents were not significantly increased during osteoblastic differentiation. Recently, it has been demonstrated that several K_V_ channels are expressed in the intracellular region of cells, such as the nucleus [68,69] and mitochondria [70]. Therefore, we consider the possibility that intracellular K_V_7.3 could be involved in the differentiation process. Moreover, during osteoblast differentiation, K_V_7.3 mRNA expression in these cells initially declined, then increased remarkably at day 14. Similar to our results, alterations of gene expressions during differentiation have been reported in studies of ion channels [26,71,72]. Variation of the pannexin *2* gene was observed in neurogenesis [71] and K_V_3.1 transcript levels were shown to oscillate during adipogenesis [26]. Unlike the variation of K_V_7.3 transcripts, expression of K_V_7.3 proteins remained at the same levels, then was augmented after day 4. Although these discrepancies between mRNA and proteins are commonly seen, the reason for this is not clear. Some reports have demonstrated that mRNA and protein expression levels are relative but not causative [73], and are affected by many factors, such as mRNA stabilization, translational modification, and protein degradation [74].

Mineralization occurs within the extracellular matrix at an end-stage of osteoblast differentiation [44,47]. Hence, extracellular matrix mineralization is used as an indicator to determine osteoblast differentiation or maturation [44]. First, blockade of K_V_ channels by TEA augmented the mineralized deposits during osteoblast differentiation of hMSCs. Although treatment with TEA substantially decreased cell viability, the overall blockade of K_V_ channels promoted extracellular matrix mineralization in hMSCs. Due to the fact that K_V_7 channels are sensitive to TEA [36,75] and there is a relationship between the M-current of K_V_7 channels and Ca^2+^ [30,48,49,50,51,52], a key factor of bone homeostasis, we examined the effect of K_V_7 channels on osteoblast differentiation by conducting MG-63 and Saos-2 osteoblast-like cells. The results showed that the inhibition of K_V_7.3 by linopirdine and XE991 produced more mineralized deposits. On the other hand, K_V_7 activation by flupirtine did not increase calcium deposits in the extracellular matrix.

Bone cell differentiation involves the distinct sequential expressions of different transcription factors and bone matrix proteins at each step [44,45]. K_V_7.3 blockade increased osterix, but did not affect Runx2, a known transcription factor that is a pivotal regulator in differentiating pluripotent stem cells into immature osteoblasts; it also induces essential osteoblast-derived matrix proteins [44]. However, the level of Runx2 expression declines with bone cell differentiation [76]. In fact, Runx2 suppresses osteoblast mineralization during bone development [65], and *in vivo* studies have demonstrated that overexpression of the Runx2 gene inhibits bone development during the maturation period [77,78]. Furthermore, Runx2 is required for the initial induction of bone matrix proteins, whereas it is not essential for maintaining them [65]. Since MG-63 cells are immature osteoblasts, the effect of the Runx2 transcription factor is not influential [62,63]. Osterix acts as a downstream signal of Runx2 and activates osteoblast marker genes, such as type 1 collagens and BSP [79,80]. ALP and OSC mRNA were also increased by suppressing K_V_7.3 channels. ALP and OSC are bone matrix proteins induced by the transcription factors of bone formation [81]. In this regard, K_V_7.3 inhibition stimulated not Runx2 but osterix, a downstream signal, to induce the levels of ALP and OSC, which promoted matrix mineralization.

Glutamate, the most common excitatory neurotransmitter, is primarily found in the central nervous system (CNS). However, glutamate signaling is not confined to the CNS; it is also found in non-neuronal tissues, including lung [82], megakaryocytes [83], pancreas [84,85,86], and bone [87,88,89,90,91]. Specifically, glutamatergic innervation exists in bones, similar to that of the CNS [89]. Glutamate is released from bone cells, in turn acting as an autologous signal within the bone environment, facilitating osteoblast proliferation, differentiation, and maturation [87,88,90,91,92]. Other studies have reported that treatment with glutamate receptor agonists, such as AMPA or NMDA, increased OSC levels, ALP activity, and mineralization of calcium deposits in bone matrix *in vitro* [87,90,91]. Moreover, *in vivo* studies have indicated that local injection of AMPA or NMDA into bone augments bone volume and mass [87].

MG-63 cells express the mRNA of various types of glutamate receptors, including NMDA receotors (NR)—NR1, NR2A, NR2B, NR2D, and NR3A—and some metabotropic receptors (mGluR) including mGluR1, mGluR2, mGluR3, mGluR4, mGluR5, and mGluR8 [93]. The mechanism of glutamate regulation is analogous to that of synapses in the CNS [89]. The exocytosis of glutamate is involved in the activity of synapsin via adjusting the phosphorylation of the ERK signal [94,95,96]. Additionally, the K_V_7.2/7.3 channel has been reported to be engaged in the phosphorylation of ERK1/2 in hippocampal neurons [97]. Hence, we studied whether ERK1/2 and synapsin proteins are responsible for glutamate exocytosis. The results showed that ERK1/2 phosphorylation was increased by the K_V_7.3 blockade on days 4 and 7, and the overall levels of synapsin by K_V_7.3 inhibition were higher than with K_V_7 activation. To further confirm that the glutamate induced by K_V_7.3 blockers can affect extracellular mineralization, glutamate receptor antagonists (CNQX and MK801) and a glutamate release inhibitor (riluzole) were co-applied with K_V_7 drugs. CNQX, MK801, and riluzole suppressed the promotive effects of K_V_7.3 blockers on mineralization. Hence, the blockade of K_V_7.3 enhanced the glutamate signals, which ultimately promoted the matrix mineralization.

Collagens are also involved in matrix mineralization during bone cell differentiation [47,98,99,100,101,102]. They accumulate in the extracellular matrix, promoting the formation of extracellular calcium deposits [47,98,101,102]. Mutations in collagen genes, especially type 1 collagens, is one of the factors that cause osteogenesis imperfecta [101,103,104]. We confirmed that the level of intracellular type 1 collagens was enhanced by the blockade of K_V_7.3 channels, whereas K_V_7 activation caused no significant difference. Thus, we concluded that the increase of type 1 collagens promoted the maturation and mineralization of the bone cell matrix.

Taken together, our findings suggest that K_V_7.3 may have potential as a regulator of osteoblast differentiation. The results indicated that inhibition of the K_V_7.3 channel led to the augmentation of osterix expression, and it also increased the extracellular glutamate release that responds to the upregulation of synapsin mediated by ERK1/2 phosphorylation. These pathways resulted in increased ALP and OSC mRNA, as well as deposition of type 1 collagen proteins, which subsequently enhanced extracellular matrix mineralization during osteoblast differentiation. Osteopenia and osteoporosis are common health problems around the world [105]. Medications for osteoporosis are generally anti-resorptive reagents acting on osteoclasts [40,106,107,108,109] and the modulators of estrogen [110], calcitonin [109,111], and parathyroid hormone [40,109] to increase blood Ca^2+^ concentration. However, side effects have been reported with drugs that have inhibitory effects on osteoclasts, such as bone fractures [112,113], skeletal pain [113,114], and increased risk of cancer [115,116]. Hormone-modulating agents also have potential adverse effects [110,111]. Therefore, advanced treatment has recently been explored, such as targeting molecular pathways [40]. In this respect, although further studies are necessary to elucidate the specific mechanisms in a living body, K_V_7.3 channel might serve as a potential therapeutic target for bone-loss-related diseases.

## 4. Materials and Methods

### 4.1. Materials

Tetraethylammonium (TEA) a non-specific K_V_ channel blocker, was obtained from Sigma-Aldrich (St. Louis, MO, USA). K_V_7 channel modulators, including flupirtine maleate, linopirdine dihydrochloride, and XE991 dihydrochloride, were purchased from Tocris Bioscience (Minneapolis, MN, USA). Riluzole, a glutamate release inhibitor, and CNQX and MK801, glutamate receptor antagonists, were obtained from Tocris Bioscience.

Rabbit polyclonal anti-K_V_7.3 antibody was obtained from Alomone Labs (Jerusalem, Israel). Rabbit polyclonal anti-ERK1/2 and phospho-ERK1/2 antibody were purchased from Cell Signaling Technology (Danvers, MA, USA) and goat polyclonal synapsin Ia/b and goat polyclonal type 1 collagen antibody were purchased from Santa Cruz Biotechnology, Inc. (Dallas, TX, USA). Horseradish peroxidase-conjugated anti-rabbit, anti-mouse antibodies were purchased from GenDEPOT (Barker, TX, USA) and the anti-goat antibody came from Santa Cruz Biotechnology, Inc.

### 4.2. Cell Culture and Osteoblast Induction

Cultured hMSCs, derived from the iliac crests of normal human donors, were purchased from Pharmicell Co., Ltd. (Seoul, South Korea). The hMSCs were maintained in the growth medium (GM) consisting of low-glucose Dulbecco’s modified Eagle’s medium (DMEM) containing 10% fetal bovine serum (FBS), 0.3 mg/mL of glutamine, 100 units/mL of penicillin, and 100 μg/mL of streptomycin at 37 °C in a humidified atmosphere of 95% air and 5% CO_2_. hMSCs at passages 4–5 were used in all experiments.

MG-63 cells and Saos-2 cells were purchased from Korean Cell Line Banks (Seoul, South Korea). MG-63 cells were cultured in GM consisting of high-glucose DMEM containing 10% FBS and 1% antibiotic antimycotic solution in an incubator at 37 °C with 95% air and 5% CO_2_. Saos-2 cells were maintained in Rosewell Park Memorial Institute (RPMI) 1640 medium with 25 mM HEPES (4-(2-hydroxyethyl)-1-piperazineethanesulfonic acid) containing 10% FBS and 1% antibiotic antimycotic solution, under the same conditions. MG-63 cells at passages 121–130 and Saos-2 cells at passages 52–61 were used in all experiments.

To induce osteoblast differentiation, 50 mg/mL of ascorbic acid (Sigma-Aldrich), 10 mM of β-glycerophosphate (Sigma-Aldrich), and 10 nM of dexamethasone (Sigma-Aldrich) were added to the maintenance GM. For osteoblast differentiation, cells were harvested using trypsin/EDTA. The detached cells were then plated onto 6-well plates at a density of 10^5^ cells/well for 24 h to allow cell attachment. On the following day, the cells were pre-incubated with flupirtine (30 μM), linopirdine (30 μM), XE991 (10 μM), riluzole (50 μM), CNQX (50 μM), and MK801 (50 μM) for 2 h, and then transferred to an osteoblast-induction medium (OM) containing these drugs and left for 14 days. The drug-containing medium was replaced twice a week. Cells in the control groups were pre-incubated with GM for 2 h, and then transferred to OM without drugs for 14 days.

### 4.3. Cell Viability Assay

An Alamar Blue assay was used to determine the cytotoxicity of TEA (10 mM) by adding 10X Alamar Blue solution (Biosource, Blue Bell, PA, USA) to each culture well, and the plates were incubated at 37 °C for 2 h. The absorbance of the plates was then measured at 570 nm, normalized to the OD values at 620 nm as a reference wavelength.

The MTT (3-(4,5-dimethylthiazol-2-yl)-2,5-diphenyltetrazolium bromide) assay was used to measure the cytotoxicity of flupirtine (30 μM), linopirdine (30 μM), and XE991 (10 μM). The concentration of these drugs was based on the EC_50_ or IC_50_ value for each drug. MG-63 cells were plated onto 96-well plates at a concentration of 10^4^ cells/well in GM or OM. The cells were treated with flupirtine, linopirdine, and XE991 for 24, 48, and 72 h, respectively. After washing the cells with Dulbecco’s Phosphate-Buffered Saline (DPBS), they were incubated with 200 μL of DPBS containing 0.5 mg/mL of MTT for 4 h. The formazans created by the viable cells were solubilized with 200 μL of dimethylsulfoxide. The absorbance of each well was measured at 570 nm.

### 4.4. RNA Extraction, RT-PCR and qRT-PCR

Total cellular RNA was extracted with RiboEx^TM^ (GeneAll, Seoul, South Korea) and DNase I (TaKaRa, Nojihigashi, Japan) according to the manufacturer’s protocol. To synthesize cDNA, 1 μg of RNA was used by M-MLV reverse transcriptase (Invitrogen, Waltham, MA, USA). Specific primers were employed for RT-PCR (Table 1). The PCR products were then electrophoresed in a 1.6% agarose gel and the expression levels of the target genes were confirmed quantitatively by real-time PCR (Applied Biosystems, Waltham, MA, USA) using SYBR^®^ Premix Ex Tag (TaKaRa). Gene expression was quantified using the comparative threshold cycles, and the relative gene expressions were compared to the ratios of the reference gene (GAPDH) threshold cycles.

### 4.5. Alizarin Red S Staining and Quantification

Alizarin Red S staining was used to determine the extent of calcium deposits, an indicator of mineralization. hMSCs were cultured in OM containing TEA for 16 days. MG-63 and Saos-2 cells were cultured in OM with K_V_7 modulators for 14 days. They were then fixed with 70% ice-cold ethanol for 1 h and stained with 2% Alizarin Red S solution, pH 4.1–4.3 (Sigma-Aldrich). After Alizarin Red S staining, the cells were dissolved in 10% cetylpyridinium chloride, and the OD values at 570 nm were analyzed for the quantification of calcium deposits.

### 4.6. Western Blot Analysis

Total cell lysates were extracted by treating the cells with a lysis buffer containing 50 mM of Tris-HCl (pH 8.0) with 150 mM of sodium chloride, 1.0% igepal CA-630 (NP-40), 0.5% sodium deoxycholate, and 0.1% sodium dodecyl sulfate (Sigma-Aldrich) and by adding 1% protease inhibitors (Sigma-Aldrich) and 10% phosphatase inhibitors (Roche, Basel, Switzerland). The cell lysates were incubated on ice for 10 min and centrifuged at 10,000× *g* for 10 min at 4 °C, and the supernatant was used for the whole-cell lysates. The bicinchoninic acid (BCA) assay was used to determine protein concentrations.

SDS-PAGE electrophoresis was conducted on 8%–12% acrylamide gels according to the size of target proteins, and the separated proteins were transferred onto the nitrocellulose membranes. Membranes were then blocked with TBST (Tris Buffered Saline, 0.1% Tween-20) containing 5% skim milk for 1 h at room temperature. Specific antibodies were added to TBST containing 5% skim milk, and the membranes were incubated at 4 °C overnight. After washing with TBST three times, the membranes were incubated with horseradish peroxidase-conjugated anti-rabbit, anti-mouse, or anti-goat antibodies for 1 h at room temperature. After washing with TBST three times, proteins were identified using enhanced chemiluminescence (ECL) solution (Advansta Inc., Adams, CA, USA).

### 4.7. Extracellular Glutamate Assay

The release of glutamate into the culture medium by MG-63 cells was measured with a glutamate colorimetric assay kit (BioVision Inc., Milpitas, CA, USA) according to the manufacturer’s procedure. To determine the release of glutamate into the extracellular medium, the cells were plated onto 6-well plates at a density of 10^5^ cells/well, followed by the same protocol of inducing osteoblast differentiation described above. On days 2 and 4, the cultured medium was collected for glutamate assay, and the remaining cells were used for the quantification of proteins. The medium used in the glutamate assay was phenol red-free DMEM.

### 4.9. Electrophysiological Recordings

MG-63 cells were detached using trypsin/EDTA, and then cells at density of 5 × 10^3^ were precipitated and suspended in the recording chamber (0.7 mL) with extracellular recording solution containing (in mM): 130 NaCl, 4 KCl, 1.8 CaCl_2_, 1MgCl_2_, 10 HEPES, and 10 glucose (adjusted to pH 7.4 with NaOH). Patch pipettes were pulled the borosilicate glass capillaries of 1.7-mm diameter and 0.5-mm wall thickness (World Precision Instruments, Sarasota, FL, USA), resulting in an open resistance ranging from 3 to 9 MΩ. The pipette internal solution (in mM) contained 110 KCl, 10 HEPES, 5 K_4_BAPTA, 5 K_2_ATP, and 1MgCl_2_ (adjusted to pH 7.2 with KOH).

The patch electrode was located on an individual precipitated cell under bright light (BX50WI, Olympus, Tokyo, Japan) with the aid of a three-dimensional hydraulic micromanipulator (Narishige, Tokyo, Japan). Ionic currents were conducted with the whole-cell voltage-clamp configuration by using Axoclamp 2B amplifier (Axon Instruments, Foster City, CA, USA). Electric signal was filtered at 1 kHz and digitized at 10 kHz using analog-digital converter (Digidata 1320A, Axon Instruments Inc., Union City, CA, USA) and pClamp software (Version 9.0, Axon Instruments Inc.). To generate current-voltage relationships, the membrane potential was held at −80 mV, test potentials ranged from −80 to +40 mV in 10 mV increments for 1.5 s. XE991 was applied into the external solution for blocking K_V_7 currents at final concentration of 50 μM.

### 4.10. Statistical Analysis

The values were presented as mean ± standard error of the mean. Student’s *t*-test was used when comparing two different groups. *p*-values of less than 0.05 were considered to be statistically significant.

## 5. Conclusions

In the present study, we identified functional K_V_7.3 channels in osteoblast-like cells and demonstrated its role in osteoblast differentiation. We confirmed that blockade of K_V_7.3 channels can promote extracellular matrix mineralization during osteoblast differentiation. The promotive effect of K_V_7.3 blockade on matrix mineralization was confirmed by (i) the augmentation of ALP, osteocalcin, and osterix transcripts and type 1 collagen proteins; and by (ii) the increase of extracellular glutamate release that responses to the upregulation of synapsin mediated by ERK1/2 phosphorylation. In conclusion, this study suggests that K_V_7.3 may be a novel regulator in osteoblast differentiation. Though further studies are required to elucidate the underlying mechanisms, our findings provide that K_V_7 channel may be one of the potential therapeutic targets in bone loss-related diseases.

## Figures and Tables

**Table 1:** Sequences of PCR primers used for RT-PCR and quantitative PCR.

Gene	Primer	Sequence	Product Size (bp)	Accession Numbers
*K_V_7.1*	Forward	CCCAAGAAGTCTGTGGTGGT	154	NM_000218
	Reverse	TGTCATAGCCGTCGACAGAG		
*K_V_7.2*	Forward	GCAAGCTGCAGAATTTCCTC	201	NM_004518
	Reverse	AGTACTCCACGCCAAACACC		
*K_V_7.3*	Forward	GGTGCAGGTCACGGAGTATT	174	NM_001204824
	Reverse	GGGCTGACTTTGTCAATGGT		
*K_V_7.4*	Forward	CTGGGCATCTCTTTCTTTGC	160	AH007377
	Reverse	GTACCAGGTGGCTGTCAGGT		
*K_V_7.5*	Forward	CGCTTTCGTTTTTCTCCTTG	207	NM_001160134
	Reverse	CGAGCAAACCTCAGTCTTCC		
*ALP*	Forward	CCTCCTCGGAAGACACTCTG	139	NM_000478
	Reverse	GCAGTGAAGGGCTTCTTGTC		
*OSC*	Forward	GACTGTGACGAGTTGGCTGA	119	NM_001199662
	Reverse	CTGGAGAGGAGCAGAACTGG		
*Runx2*	Forward	CACCGAGACCAACAGAGTCA	95	NM_001015051
	Reverse	TGATGCCATAGTCCCTCCTT		
*Osterix*	Forward	GCCAGAAGCTGTGAAACCTC	161	AF477981
	Reverse	GCTGCAAGCTCTCCATAACC		
*GAPDH*	Forward	CTCTGCTCCTCCTGTTCGAC	112	NM_002046
	Reverse	ACGACCAAATCCGTTGACTC		
